# New Insights in Radiotherapy

**DOI:** 10.3390/biomedicines10081931

**Published:** 2022-08-09

**Authors:** Carlos Martínez-Campa

**Affiliations:** Department of Physiology and Pharmacology, School of Medicine, University of Cantabria and Instituto de Investigación Sanitaria Valdecilla (IDIVAL), 39011 Santander, Spain; carlos.martinez@unican.es

This Special Issue of *Biomedicines*, entitled “New insights in Radiotherapy”, compiles insightful reviews on the state of the art on different aspects of radiation therapy, and also collects high-quality research articles highlighting the latest advances in the use of ionizing radiation to treat a variety of specific diseases, including cancer, either with curative of palliative purposes. Radiotherapy uses waves of high energy, usually ionizing radiation. At the cellular level, it damages the DNA, inducing double-strand breaks, hence disrupting cell growth and mitosis. Thus, radiation prevents, or at least slows down, the spread of the disease. However, unfortunately, radiation affects both cancer cells and normal surrounding tissues, the evolution of new radiotherapy techniques has improved dose delivery whilst reducing the dose to the surrounding normal cells.

This Special Issue, entitled “New Insights in Radiotherapy”, includes both reviews and original research on advances in radiation cancer treatments. Clement et al. have addressed the effectiveness of a novel nanoparticle drug formulation for radiodynamic therapy (RDT). RDT is based on the generation of reactive oxygen species (ROS) at the lesion site when the interaction between X-rays and a photosensitizer drug (PS) is take into account. To overcome the problem of hypoxia (resulting in a low availability of oxygen for generating sufficient amounts of ROS), the authors have synthesized poly (lactic-co-glycolic acid) (PLGA) nanoparticles (NPs) co-loaded with a PS drug verteporfin (VP), and the clinically approved oxygen-carrying molecule, perfluorooctylbromide (PFOB). PLGA–VP–PFOB nanoconstructs (NCs) significantly increased the ROS production and showed a high efficiency in killing human pancreatic tumor cells grown both in monolayer cultures and in a 3D model of pancreatic cancer liver metastasis, thus suggesting that PLGA–VP–PFOB is a promising agent for the RDT of deep-seated hypoxic tumors [1].

Nicosia et al. focused their research on exploring the effect of magnetic fields (MFs) as an enhancer of radiation effects in patient-derived organoids (PDOs) derived from pancreatic ductal adenocarcinoma (PDAC), which is a kind of cancer highly refractory to radiotherapy alone or combined with chemotherapy. In their model, a high-intensity static magnetic field increased radiation effectiveness. Those PDOs treated with both MF and radiation were significantly smaller in size and showed an increase in cell death as compared with those treated in monotherapy with radiation alone, suggesting that the clinical application of magnetic-enhanced radiotherapy should be explored [2].

External beam radiotherapy (EBRT) delivers dose fractions to gradually destroy a tumor while minimizing the side effects in neighboring normal tissues. In their study, Moreau et al. investigated how deep reinforcement learning (DRL) approaches can learn from a model of a mixture of tumor and healthy cells. Two different algorithms were trained, with different preference functions, and the results suggest that initiating treatments with larger doses per fraction and subsequently gradually reducing them might be more effective than the standard approach of using a constant dose per fraction [3].

Karsten et al. performed a retrospective study on patients in different stages of follicular lymphomas, examining the role of ^18^F-fluorodeoxyglucose positron emission tomography/computed tomography (^18^F-FDG-PET-CT). Their study showed that involved field radiation combined with immunotherapy is a valuable therapeutic tool for the treatment of early-stage nodal and extranodal follicular lymphomas. ^18^F-FDG-PET-CT is an optimal approach for staging, especially in early-stage follicular lymphomas, which enables the identification of patients with excellent prognosis by radiotherapy [4].

Ionizing radiation can cause deleterious effects in non-small-cell lung cancer (NSCLC) patients, such as metastasis, thus leading to poor prognosis. Kim et al. have studied the effect of dendrobine, a plant-derived alkaloid, as an enhancer of ionizing radiation radiotherapy. In a mouse model of ionizing-radiation-induced metastasis, dendrobine effectively inhibited NSCLC cell migration and invasion through suppression of the expression of sulfatase2 (SULF2), involved in the activation of the SULF2/β-catenin/STAT3/SOD2/Bcl-X_L_ signaling pathway, which eventually promotes tumor cell invasion in an Src-dependent pathway, suggesting that dendrobine has value as a potential therapeutic enhancer to mitigate the malignant effects of radiotherapy in NSCLC [5].

Alonso-González et al. tested the effects of melatonin, a pineal-gland-synthesized hormone with peak levels occurring at night, in estrogen-dependent breast cancer cells subjected to radiation. Melatonin potentiated some of the therapeutic effects of radiation, such as its anti-proliferative effect, gene expression, inhibition of the Akt pathway, miRNA expression, and new vascularization in vivo, but also counteracted some of the undesirable deregulation of both gene and miRNA expression induced by radiotherapy; in some cases, dangerous changes with the potential to promote survival, proliferation, invasion, epithelial-to-mesenchymal transition, metastasis, and resistance, results that strongly suggest the potential role of melatonin as a radiosensitizer in breast cancer treatment [6].

This Special Issue also includes reviews exploring different aspects of radiation treatments. Alonso-González et al. have reviewed the state of the art of the role of melatonin both as a radio-sensitizer, enhancing the response of tumor cells to radiation, and as a radio-protector, reducing the deleterious effects triggered by radiotherapy in normal surrounding tissues. The radiosensitization of some types of cancer by melatonin is related to the upregulation of p53, the modulation of several enzymes involved in estrogen biosynthesis, reduction in the capability of cancer cells to repair DNA damage and enhancement of the DNA damage induced by radiation, enhancement of anti-angiogenic actions, and an increase in apoptosis promoted by radiation. Melatonin also has effects on the tumor microenvironment, modulating the interactions between epithelial cancer cells and pre-adipocytes. In summary, all the information compiled suggest melatonin to be a promising radiosensitizer agent that should be implemented to improve individual results in cancer patients [7].

Obrador et al. reviewed the state of the art on radioprotectors (which directly reduce the DNA damage caused by radiation) and radiomitigators (which protect against toxicity even after radiation has been applied). There is currently a need for the development of efficacious countermeasures to protect patients from the harmful effects of radiotherapy, but, presently, no new chemicals have been approved by the Food and Drug Administration (FDA) as radiation countermeasures for acute radiation syndrome (ARS). The authors discuss the new molecules currently under development and the difficulties in finding ideal radioprotectors or radiomitigator agents, because the injury mechanisms triggered by radiation are not completely understood [8].

The cytoskeleton is a structure whose main function is to provide structural support, thus helping cells to maintain their internal organization and their shape. Radiation-induced alterations in the cytoskeletal architecture can lead to a loss of adhesion, as well as increased motility and invasiveness. In their review, La Verde et al. compiled the results of several studies which have addressed the effects of radiation on the cytoskeleton, finding that there is a great heterogeneity of results depending on the cell line tested, the different time frames analyzed, the cell model systems, and the microenvironmental conditions, such as the extracellular matrix, which seems to have a central role in controlling cell behavior. The authors point to the importance of standardizing models and experimental conditions in the search of an optimized protocol [9].

Therapeutic responses to different anti-cancer therapies can be evaluated by measuring the diameter of tumors and quantitative parameters of 2-[^18^F] fluoro-2-deoxy-d-glucose positron emission tomography (FDG-PET). In their review, Oriuchi et al. analyzed current knowledge on the tumor microenvironment, focusing on cancer cell metabolism, cancer stem cells, chemokines and their receptors, and immune mechanisms, all of which are targets of therapy. Taking into account the heterogeneity of tumors and the individual variation in different treatments, molecular imaging techniques such as FDG-PET may help to address therapeutic responses and toxicity evaluations to generate valuable information for the benefit of novel anti-cancer therapy. PET imaging plays can play a crucial role in the assessment of individual therapeutic effectiveness towards the development of personalized therapeutic strategies within the framework of precision medicine [10].

In summary, the 10 contributions accepted in this Special Issue highlight the latest advances on ionizing radiation and the perspectives on new strategies pursuing the enhancement of radiotherapy efficacy and imaging techniques as well as the utilization of novel molecules acting as radiosensitizers, radioprotectors, and radiomitigators.
